# Tailoring Rheumatoid Arthritis Visit Timing Based on mHealth App Data: Mixed Methods Assessment of Implementation and Usability

**DOI:** 10.2196/60854

**Published:** 2025-04-21

**Authors:** Robert S Rudin, Leah M Santacroce, Ishani Ganguli, Daniel H Solomon

**Affiliations:** 1 RAND, 20 Park Plaza, Boston, MA, 02116, United States, 1 6173382059; 2 Division of Rheumatology, Inflammation, and Immunity, Brigham and Women's Hospital, Boston, MA, United States; 3 Division of General Internal Medicine and Primary Care, Brigham and Women's Hospital, Boston, MA, United States; 4 Harvard Medical School, Boston, MA, United States; 5 Division of Pharmacoepidemiology and Pharmacoeconomics, Department of Medicine, Brigham and Women's Hospital, Boston, MA, United States

**Keywords:** mobile health, patient-reported outcomes, visit timing, visit frequency, rheumatoid arthritis, rheumatology

## Abstract

**Background:**

Visits to medical subspecialists are common, with follow-up timing often based on heuristics rather than evidence. Unnecessary visits contribute to long wait times for new patients. Specialists could enhance visit timing and reduce frequency by systematically monitoring patients’ symptoms between visits, especially for symptom-driven conditions like rheumatoid arthritis (RA). We previously designed an intervention using a mobile health (mHealth) app to collect patient-reported outcomes (PRO). One of several aims of the app was to assist rheumatologists in determining visit timing for patients with RA. The intervention did not reduce visit frequency.

**Objective:**

To explore possible reasons for the lack of association between the intervention and visit frequency, we describe app usage, assess usability, and identify barriers and facilitators for using between-visit PRO data to reduce visits when patients’ symptoms are stable.

**Methods:**

We analyzed patients’ use of the app by reporting adherence (percent of PRO questionnaires completed during the 12-month study) and retention (use in the last month of the study). To examine rheumatologists’ experiences, we summarized views of the electronic health record (EHR)–embedded PRO dashboard and EHR inbox messages suggesting early or deferred visits. We assessed app usability using the interactive mHealth App Usability Questionnaire for Ease of Use and Usefulness for patients and the System Usability Scale for rheumatologists. We assessed rheumatologist-level effects of intervention usage using Kruskal-Wallis rank sum and equality of proportion tests. We identified barriers and facilitators through interviews and surveys.

**Results:**

The analysis included 150 patients with RA and their 11 rheumatologists. Patients answered a median of 53.3% (IQR 34.1%-69.2%) of PRO questionnaires; this proportion varied by rheumatologist (range 40.7%‐67%). Over half of the patients used the app during the final month of the study (56%, range 51%‐65%, by rheumatologists); the median number of months of use was 12 (IQR 9-12). Rheumatologists viewed the dashboard 78 times (17.6% of 443 visits) with significant differences in viewing rates by rheumatologist (range 10%‐66%; *P*<.01). There were 108 generated messages sent to rheumatologists suggesting a deferred visit (24.4% of 443 visits) with significant differences in message counts received per visit by rheumatologist (range 10.8%‐22.6%; *P*=.03). Rheumatologists’ reported barriers to offering visit deferrals included already scheduling as far out as they were comfortable and rescheduling complexities for staff. Based on 39 patient interviews and 44 surveys, patients reported 2 main barriers to app usage: questionnaire frequency not being tailored to them and reduced motivation after not discussing PRO data with their rheumatologist. A total of 5 interviewed patients received the option to defer their visits, of which 3 elected to defer the appointment and 2 chose to keep it.

**Conclusions:**

While an mHealth app for reporting RA PROs was used frequently by patients, using these data to reduce the frequency of unneeded visits was not straightforward. Better engagement of clinicians may improve the use of PRO data.

## Introduction

Visits to medical specialists represent a substantial component of health care services. About half of patient visits to physicians in the United States are to specialists, totaling roughly 500 million visits per year at a cost of billions of dollars [1,2]. The current approach to care delivery inefficiently allocates specialty care resources: visits are scheduled largely in preset intervals (eg, 3, 6, or 12 months) that are not evidence-based and have a weak relationship to clinical need [3,4]. Sometimes these schedules are required for insurance purposes, that is, to have medications covered. Evidence for optimal visit timing is scant [3,5]. As a result, specialists’ schedules fill with potentially unneeded visits, creating inefficiencies, wasteful spending, and patient burdens [6], and contribute to longer wait times for other patients who need specialist care; these access issues are especially pronounced for rural, minority, and other vulnerable populations [7].

We previously designed a mobile health (mHealth) intervention to improve patient care in rheumatology [8]. It was well accepted by patients, and we became interested in whether one additional value of the app could be to reduce visit follow-up frequency when clinically appropriate [8,9]. The rationale was that a reasonable proportion of visits were for stable patients where remote symptom reporting could help clinicians defer visits. We focused on rheumatology because this specialty has a great need for optimizing visit timing due to projected workforce shortages in the next decade [10]. Furthermore, prior works suggest a potential for reducing visit frequency, in part, because a substantial number of visits, for rheumatoid arthritis (RA) in particular, do not result in a change in treatment, suggesting limited clinical use [8,11-13]. The mHealth intervention involved patients using a smartphone app to record their symptoms using patient-reported outcome (PRO) questionnaires; based on these data, their rheumatologists received notifications that either suggested the need for an earlier visit if symptoms were acutely worsening or the potential to defer a scheduled visit if symptoms were stable (see Methods for details).

Our initial clinical trial of this intervention found no association with reduced visit frequency. To explore possible reasons for this finding and inform further intervention development, we examined quantitative and qualitative data on patient and rheumatologist engagement and data from surveys and interviews. We describe the app intervention usage, assess usability, and identify possible barriers and facilitators to improve visit follow-up frequency using between-visit PRO data.

## Methods

### Setting and Study Population

The prior study was conducted at 2 rheumatology practices affiliated with Brigham and Women’s Hospital, which is part of Mass General Brigham, a large academic medical center in Boston, MA, United States. All clinics within this health system share an electronic health record (EHR) system (Epic Systems, Inc). The intervention was implemented by engaging 11 clinically focused rheumatologists who previously provided input on the intervention design [8,9]. As described previously, we identified eligible patients by searching upcoming patient visit schedules for the 11 rheumatologists [8,9]. We used prior diagnosis codes to identify patients with RA. Only established patients were included, and therefore all prescheduled visits were follow-ups. We recruited these patients via patient portal messages, phone calls, and in-person prior to scheduled visits.

### mHealth Intervention

The intervention was designed with extensive input from patients and rheumatologists through several iterations as part of a user-centered design process [8,9,14,15]. Briefly, the intervention included a patient-facing mHealth app that used push notifications to prompt patients to complete one of four types of validated PRO questionnaires regarding pain interference, function, fatigue, and RA disease activity [8]. Each questionnaire was repeated every 8 days in a staggered fashion so that patients completed one questionnaire every 2 days. Rheumatologists were able to view all results within the patient’s EHR chart. In addition, the PRO data could trigger 3 types of EHR inbox messages to the rheumatologists. First, all rheumatologists received a message that data were available for viewing 48 hours before a visit with a patient who had entered PRO data. Second, if PRO data suggested several weeks of worsening symptoms, the rheumatologist received a message with a suggestion to offer an earlier visit. Finally, if PRO data suggested stable symptoms during the visit interval from the prior visit until 2 weeks prior to a scheduled visit, rheumatologists received a message suggesting that the patient be offered a deferred visit. The specific logic that determined when these messages are sent has been described previously [8].

### Study Design and Data Sources

As noted, this initial trial of the mHealth app found no change in visit frequency attributable to the PRO data and EHR inbox messages [9]. We sought to explore potential reasons for these findings and inform further intervention development by (1) describing use of the mHealth app by patients and use of the PRO data by rheumatologists, (2) assessing mHealth app and EHR-embedded PRO data usability, and (3) identifying barriers and facilitators. Patient and rheumatologist perspectives were assessed through surveys during and after the study period. Data sources included the EHR (for demographic and clinical characteristics of study patients and visit timing), mHealth app and dashboard usage logs (for patient adherence and retention, rheumatologist views of the PRO data, and messages sent suggesting early or deferred visits), interviews with patients (for barriers and facilitators), and surveys of patients and rheumatologists (for usability and barriers and facilitators).

For analyses of intervention usage and usability, rheumatologists with 15 or more patients in the study were analyzed as individuals, and the rest were aggregated. Because only one of the instances in which an earlier visit was suggested resulted in an earlier visit, we focused the analysis primarily on aspects related to deferred visits [9].

### Intervention Usage Analysis

We used descriptive statistics to summarize patient adherence and retention rates with the mHealth app. We used the Kruskal-Wallis rank sum test to assess differences in median patient PRO completion percentage across rheumatologists. We used descriptive statistics to summarize volumes and timing of EHR inbox messages sent to the rheumatologists suggesting the potential for an early or deferred visit and view of the EHR-embedded PRO data. For both measures, we tested for equality of proportions across rheumatologists.

### Usability Analysis

We summarized results from mHealth App Usability Questionnaire (MAUQ) items, which were administered to patients after they completed the study [16]. The MAUQ is a validated instrument measuring mHealth app usability. Specifically, we used the interactive versions of the ease of use and satisfaction (MAUQ_E) and usefulness (MAUQ_U) subscales of the questionnaire because they were most relevant to this intervention. The MAUQ scales range from 1 (worst, ie, strongly disagree with all questions) to 7 (best, ie, strongly agree with all questions), with 4 meaning neutral.

For rheumatologists, we summarized the results of the System Usability Scale (SUS) administered after all the rheumatologists’ patients had completed the study [17,18]. The SUS is a widely used usability measure with a range from 0 (worst) to 100 (best), with an average score across evaluations of 68‐70.

### Barriers and Facilitators Analysis

We invited patients who had received a suggested early or deferred visit to participate in an interview. We conducted those interviews virtually and transcribed the recordings. Using conventional content analysis, one research team member (RSR) created a codebook and summarized the results using the framework method, and a second research team member (DHS) reviewed a portion of the results to confirm findings [19,20]. We used similar methods to analyze free-text responses from patient survey results administered at study completion. When presenting qualitative results from interviews, we used “some” to indicate a finding was present in fewer than 5 responses, “many” to denote greater than 5 responses, and “most” to denote greater than half of the responses.

For rheumatologists, we summarized data from a survey administered after study completion about how often they offered patients the option to defer a visit and the reasons for not offering that option.

All quantitative analyses were conducted in R (version 4.2.2; R Foundation for Statistical Computing).

### Ethical Considerations

This study was reviewed and approved by the institutional review board at Mass General Brigham (number 2021P000790). All written and audiovisual data (which were not anonymized) were stored on secure servers accessible only to the research team. Written consent was obtained for all patients to participate in the trial and for the use of their medical data in analyses, verbal consent was obtained for interviews, and consent information was presented before all electronic survey questions. Participants were compensated with a gift card for the trial and a US $25 check for the interviews.

## Results

### Participants

Characteristics of the 150 intervention patients included in the study are shown in Table 1. Patients were mostly female and White.

**Table 1.** Characteristics of patients using the mHealth app for rheumatoid arthritis (N=150).

Characteristics	Values
Sex, n (%)	
Female	125 (83.3)
Male	25 (16.7)
Age (years), median (IQR)	62 (52-69)
Race, n (%)	
Asian	4 (2.7)
Black	5 (3.3)
White	136 (90.7)
Declined or others	5 (3.3)
Seropositivity status, n (%)	
Missing	36 (24)
0	39 (26)
1	75 (50)
CRP^a^^,^^b^, median (IQR)	1.4 (0.7‐4.5)
DMARD^c^^,^^d^ use, n (%)	
csDMARD^e^	93 (62)
btsDMARD^f^	90 (60)
Other medications, n (%)	
NSAIDs^g^	30 (20)
Corticosteroid	55 (36.7)
Opioids	25 (16.7)
Comorbidities, n (%)	
Hypertension	26 (17.3)
Obesity	15 (10)
Depression	7 (4.7)
Osteoporosis	19 (12.7)

a CRP: C-reactive protein.

b Two values were missing for CRP.

c DMARD: disease-modifying antirheumatic drug.

d It is common for patients to use more than 1 DMARD.

e csDMARD: conventional synthetic disease-modifying antirheumatic drug.

f btsDMARD: biologic or targeted synthetic disease-modifying antirheumatic drug.

g NSAID: nonsteroidal anti-inflammatory drug.

### Intervention Usage

**Figure 1.**
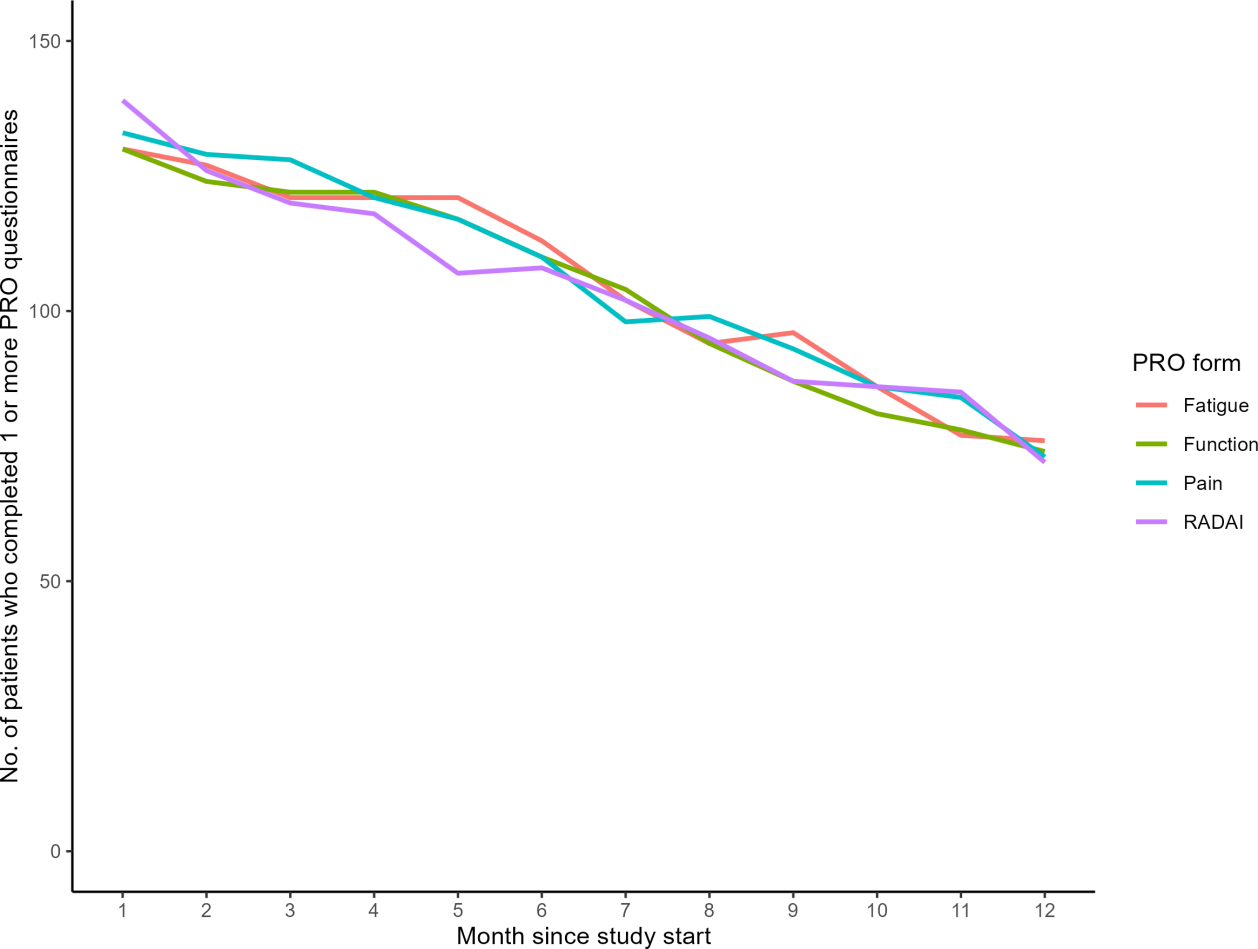
Patient questionnaire completion rates within the rheumatoid arthritis app over the 12-month study period. PRO: patient-reported outcome; RADAI: Rheumatoid Arthritis Disease Activity Index.

Four rheumatologists each had at least 15 patients in the intervention and were analyzed as individual observations; the other 7 rheumatologists were aggregated and treated as one observation. The median percentage of PRO questionnaires completed was 53.3% across all patients (Table 2). This completion rate varied by rheumatologists, ranging from 40.7% (the rheumatologist with the most patients) to 67% (the rheumatologist with the third most patients). Differences in completion rates between all rheumatologists or groups were not statistically significant based on the Kruskal-Wallis rank sum test (*P*=.50). The median number of months patients answered questionnaires was 12 (IQR 9-12), with little variation by rheumatologist; 56% (84/150) of patients completed at least one questionnaire in the final month of the study. See app usage by rheumatologists in Table 2 and over time in Figure 1.

**Table 2.** App usage by rheumatoid arthritis patients grouped by their rheumatologist.

Rheumatologist^a^	Patients, n	Patient visits, n	Percent PRO^b^ questionnaires completed,^c^ median (IQR)	Months with answered questionnaires,^d^ median (IQR)	Patients who completed at least 1 PRO in the final month of the study, n (%)
All rheumatologists	150	443	53.3 (34.1-69.2)	12 (9-12)	84 (56)
Four rheumatologists with the greatest volume of study patients					
1st highest	51	148	40.7 (25.8-63.7)	11 (8-12)	26 (51)
2nd highest	25	68	48.9 (34.1-64.8)	12 (9-12)	13 (52)
3rd highest	20	53	67 (49.5-78.6)	12 (11-12)	13 (65)
4th highest	15	49	46.7 (44-61)	12 (9-12)	8 (53)
All other rheumatologists					
5th-11th highest	39	125	58.8 (47.3-72)	12 (11-12)	24 (62)

a Ordered by volume of study patients who receive care from that rheumatologist.

b PRO: patient-reported outcome.

c The total possible PRO questionnaires offered were 182.

d Number of months within the 12-month study in which patients answered at least 1 PRO questionnaire.

There was variation by rheumatologist in the percentage of their received messages that suggested a deferred visit, ranging from 18.9% of patient visits (the rheumatologist with the most patients) to 37.7% (the rheumatologist with the third most patients) (Table 3). In a 6-sample test for equality of proportions, there was a statistically significant difference in the percent of deferred visit suggestions received by rheumatologists (*P*=.03). There were no significant differences among early visits. For rheumatologist usage of the PRO data as measured by the number of views, there was variation by rheumatologist ranging from 10.1% of patient visits (rheumatologist with the most patients) to 66% (rheumatologist with the third most patients). In a 6-sample test for equality of proportions, the difference was statistically significant (*P*<.01), which means that at least one value (number of views of PRO data per patient visit) differed significantly from the others.

**Table 3.** Usage of rheumatologist-facing intervention information generated by the rheumatoid arthritis app, available within the electronic health record.

Rheumatologist^a^	Patients, n	Patient visits, n	Messages sent to rheumatologist suggesting visit deferral, n (%)	Messages sent to rheumatologist suggesting early visit, n (%)	Rheumatologist views of PRO^b^ data in EHR^c^, n (%)
All rheumatologists	150	443	108 (24.4)	31 (6.9)	78 (17.6)
Four rheumatologists with the greatest volume of study patients					
1st highest	51	148	28 (18.9)	8 (5.4)	15 (10.1)
2nd highest	25	68	13 (19.1)	5 (7.4)	11 (16.2)
3rd highest	20	53	20 (37.7)	5 (9.4)	35 (66)
4th highest	15	49	18 (36.7)	3 (6.1)	7 (14.3)
All other rheumatologists					
5th-11th highest	39	125	29 (23.2)	10 (8)	10 (8)

a Ordered by volume of study patients who receive care from that provider.

b PRO: patient-reported outcome.

c EHR: electronic health record.

### Usability

**Figure 2.**
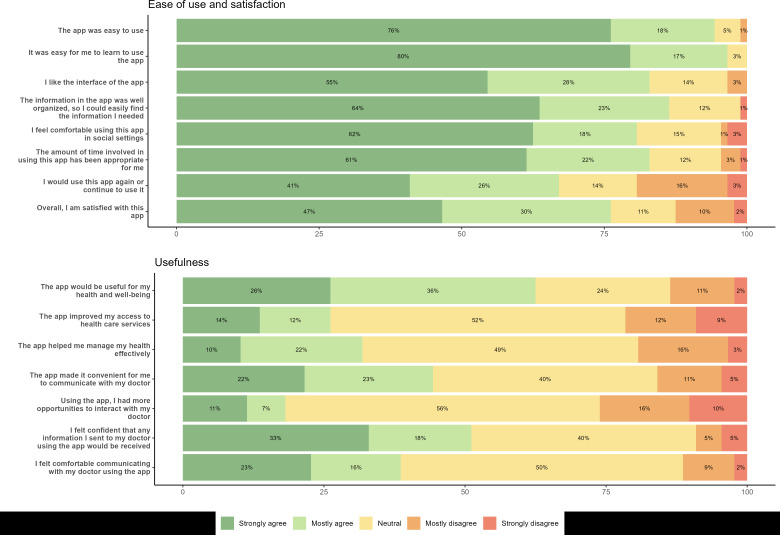
Rheumatoid arthritis app usability as measured by the mHealth App Usability Questionnaire for Ease of Use (MAUQ_E) and Usefulness (MAUQ_U) for clinically integrated apps, administered at the completion of the 12-month study.

Of the 150 intervention patients, 88 completed the MAUQ surveys with a median MAUQ_E of 6.3 (IQR 5.6-6.9) and median MAUQ_U of 4.8 (IQR 4.1-5.6) (Table 4 and Figure 2). Based on Figure 2, most patients found the app easy to use across all questions. However, the usefulness of the app was less clear for patients. Most patients agreed that the app “would be useful for my health and well-being,” but about half of patients were neutral about its usefulness on the other questions. There was a small range across rheumatologists of the aggregated MAUQ_E (6.2‐6.9) and a more substantial range for the MAUQ_U (4.6‐6.3).

Of 11 participating rheumatologists, 10 completed the SUS to assess the EHR-embedded dashboard and reported a mean of 68.25 (range 45‐82.5). (The average score for the SUS across a wide range of apps is 68 (SD 12.5) [21].)

**Table 4.** Patient usability scores of the rheumatoid arthritis app at the completion of the 12-month study.

Rheumatologist^a^	Patients, n	MAUQ_E^b^, median (IQR)	MAUQ_U^b^, median (IQR)
All rheumatologists	88	6.3 (5.6-6.9)	4.8 (4.1-5.6)
Four rheumatologists with the greatest volume of study patients			
1st highest	31	6.2 (5.5-6.6)	4.6 (3.4-5.3)
2nd highest	17	6.3 (6.2-7.0)	4.6 (4.2-5.0)
3rd highest	12	6.7 (6.4-7.0)	5.2 (4.3-6.3)
4th highest	4	6.9 (6.2-7.0)	6.3 (5.7-6.6)
All other rheumatologists			
5th-11th highest	24	6.3 (5.6-6.9)	4.8 (3.8-5.3)

a Ordered by volume of study patients who receive care from that provider.

b The mHealth App Usability Questionnaire for Ease of Use (MAUQ_E) and Usefulness (MAUQ_U) were rescaled from 1‐5 to 1‐7 (worst to best) to be consistent with their standard use, with 1 indicating strongest disagreement with all questions and 7 indicating strongest agreement with all questions.

### Barriers and Facilitators

We interviewed 39 patients shortly after their responses triggered a message to their rheumatologist suggesting a possible early or deferred visit. We also assessed free text comments from 44 patients who completed the final study survey. Facilitators and barriers are summarized in Table 5. The most cited benefit of the intervention mentioned by patients was that the app improved awareness of their symptoms: “I really enjoyed using this app and felt like it gave me a great way to check in with myself on my symptoms and take a pulse on how my RA was” [Patient 78]. Patients described benefits both from being aware of when their symptoms were worse and from recognizing when they were feeling well, such as when a medication change was working (“I’m in the green every time, it’s amazing!” [Patient 141]).

**Table 5.** Facilitators and barriers to rheumatoid arthritis patient engagement by intervention component based on interviews with users and open-ended survey responses.

Intervention component	Facilitators	Barriers
PRO^a^ questionnaires	Increased awareness of symptoms was perceived as a benefit; graph of responses helped assess the impact of medications	Frequency: Too often for those with well-controlled or stable symptoms, and too little for those with changing symptoms, reduced motivation to complete questionnaires Type: Questions did not always capture the nature of symptoms; many wanted to record free text notes Timing: Some had trouble remembering symptoms during the prior 8 days
Discussing PRO scores with rheumatologists	Discussion motivated patients to continue using the app and be aware of their symptoms	Lack of discussion reduced motivation to continue using the app
Offers for deferred visits	Perceived time saved from avoiding unneeded visits was viewed positively	Lack of knowledge that the app would inform visit timing reduced motivation to use the app; perception that laboratory testing needs would dictate visit timing impeded the use of deferrals; receiving care from multiple rheumatologists complicated visit deferral decisions; living in different locations during the year reduced opportunities for visit deferrals; concern about the ability to find a visit slot in a suitable time frame reduced interest in visit deferrals
Offers for early visits	Potential for early visits helped make patients feel looked after; patients trust in rheumatologist’s judgment made them likely to accept an earlier visit	Lack of clarity over whether symptoms were RA-related^b^ caused confusion over whether an early visit was needed
User experience with technology	App was viewed as easy to use	Technical issues, the need for questionnaires to be available earlier in the morning, and the lack of SMS notifications were challenges to using app

a PRO: patient-reported outcome.

b RA: rheumatoid arthritis.

Although most of the patients we spoke with did not discuss the app with their rheumatologists, many did, and most of those patients were appreciative and motivated to use the app because of it: “She said ‘well I can see on this graph’...it was definitely comforting because I’m in the good part...so it was good she brought it up” [Patient 28]; “I did discuss the app data with my doctor...It was very helpful to review them with her” [Patient 180]. In a few cases, patients described examples in which decisions regarding medications were made based on discussion of the app data. In 2 cases, a patient described how the app data helped inform the decision during a visit to further increase visit intervals. Some of the patients who discussed the app data with their rheumatologists said the app added little to their care: “I was cognizant that [my doctor] looked at the app and has referred to it in discussing my condition, but she is a sufficiently good and attentive communicator that I didn’t notice much in the way of changed communications with her (which I considered excellent to begin with)” [Patient 145].

However, many patients were not aware that their rheumatologist has access to or viewed the data: “My doctor and I have never discussed the use of this app. I’m not even sure she knows about the app” [Patient 144]. This reduced motivation to use the app: “I was disappointed that the app was not used at all, really, in my care. I felt that the information just went into cyberspace and was never heard from again” [Patient 119]; “I don’t see any connection between the app and the care I receive with my particular rheumatologist...I found it interesting that she didn’t bring it up so that gave me the message that she doesn’t find it helpful in her decision making” [Patient 30]. One patient noted a disconnect between her impression of the primary purpose of the app (for her rheumatologist to oversee her PRO responses and reach out if needed) and her rheumatologist’s view of the app’s main purpose (for patient self-management).

Of the 5 interviewed patients who reported receiving a call offering them the option to defer their visit because of stable symptoms they reported in the app, 3 accepted the offer to defer the visit: “Not having to take the trip down to Boston from Maine, as long as [my doctor] thought was fine, we said okay and we rescheduled it [from September until November]” [Patient 200]. One of those patients had a symptom flare shortly after electing to defer the visit: “the following week I had a bad rheumatoid attack and I thought ‘oh shoot I should have kept that appointment’ but I think it was okay” [Patient 190]. Two decided to keep their appointment: “when she called me I was in the middle of...about 5 different joints that were inflamed and so I said to her that I’d like to keep the visit” [Patient 145]. When asked about the potential to receive such a call, almost all patients interviewed had a positive view: “If I’m feeling well and it’s just kind of a check-in, then I wouldn’t need to drive into Boston for it” [Patient 70]; “I’d do a cartwheel...it is such a hassle [to drive into Boston]...traffic can be brutal” [Patient 141].

The only patient we interviewed who received a call from the clinic offering them the option for an earlier visit based on their PRO data believed that they did not need to come in early for their RA. When asked about the potential to receive such a call, almost all patients interviewed had a positive view: “when you have to call in...you’re hesitant to do that because you know you’re always wondering ‘oh is this something that’s just going to pass in a little while’...whereas if you guys could actually see the graph changing and call...it is comforting” [Patient 28]; “I think that would be great!...I’d feel like I’m not just spinning the wheel for nothing, answering the questions” [Patient 70]. However, a few believed they were already in good communication with their rheumatologist and would have contacted them if they needed help: “Very likely I would’ve already been there...I would’ve already thought to give her a call and let her know I’m not feeling great” [Patient 30].

About half of patients interviewed or who provided free text comments on the survey mentioned challenges related to the app questionnaire: some found them too frequent (“I did not see the value and found the number of surveys excessive and as I am in remission” [Patient 84]); some found them not frequent enough (“Every day would be better than every other day because you know if you do it every day then you know when you miss a day” [Patient 103]); and some found them inadequate for capturing their relevant symptoms (“This app did NOT capture the total wellbeing of the patient. It is strictly about joints. My RA involves my lungs” [Patient 3]).

Of the 11 rheumatologists, 10 completed the final survey (Table 6). Responses showed that the major barrier for rheumatologists not offering deferred visits was that they were already scheduling patients out as far as they were comfortable with. The complexity of rescheduling visits and the need for patients to come in for labs anyway were also notable barriers.

**Table 6.** Rheumatologist survey of reasons for not offering patients the option to defer a visit, administered after completion of the 12-month study.^a^

Reasons for not offering patients with stable PROs^b^ the option to defer a visit^c^	Top ranked, n^d^	In top three, n^d^	Indicated at least once
I am too busy to spend time deciding if the visit could be delayed	0	1	Yes
Most of my patients should come in even if their PROs are stable	0	1	Yes
Most of my patients need to come in for labs anyway	1	5	Yes
Rescheduling visits makes scheduling more complicated for me and my staff	2	6	Yes
Rescheduling visits increases my workload by removing easier visits	0	0	No
I am already scheduling patients as far out as I am comfortable with	3	8	Yes
If the open slots from a delayed visit are not filled, there will be lost revenue	0	0	Yes
I do not trust the PRO data to inform the decision to delay a visit	0	0	No
Delaying a visit could complicate prior authorizations	0	2	Yes
My time needed to determine if the visit could be delayed and not reimbursed	0	1	Yes
Other^e^	0	0	Yes

a N=10 responses; 1 rheumatologist had left the clinic, and all others responded.

b PRO: patient-reported outcome.

c Exact wording for questions: You received in-basket messages suggesting delayed visits during the study about patients with stable PROs who had visits scheduled in 2 weeks. The messages suggested that you consider asking your assistant to call the patient and give them the option to delay the visit. (1) Of these in-basket messages, please estimate how many for which you gave the patient the option to delay the visit (choose 1 response): none, some, all. (2) For patients for whom you did not offer a delayed visit, what do you think were the primary reasons? Rank all factors that influenced you (1=most influential and so on). (3) Please add any other thoughts you have about offering patients the option to delay a visit based on their PROs. (Write as much or as little as you’d like.)

d “Top ranked” means the respondent listed the item as their first choice. “In top three” means the respondent listed the item as their first, second, or third choice. Some respondents did not rank their responses numerically but rather selected options using an ‘x’—we did not count those in the “top ranked” and only included them in the “top three” if they selected three or fewer.

e One rheumatologist wrote, “some patients seem to want to come in anyway, even if they don’t have to come.”

## Discussion

### Principal Results

Our examination of intervention usage, usability, and barriers and facilitators provides some potential reasons—from both rheumatologist and patient perspectives—for why our prior study did not find an effect of an intervention designed to improve visit follow-up frequency.

From the rheumatologist perspective, although usability of the EHR-embedded dashboard was satisfactory, use of the dashboard varied substantially across rheumatologists, with most using it in a small percentage of visits. Many participating rheumatologists did not offer patients deferred visits because they were not comfortable increasing visit intervals any further, believed rescheduling visits was complicated for them and their staff, or allowed the laboratory testing schedule to determine visit timing.

From the patient perspective, most continued to use the app in the final month of the study and found the app easy to use as measured by the MAUQ_E. The MAUQ_U scores were less favorable, suggesting that patients found the app less useful than easy to use. Relatively few disagreed that it was useful, but many were neutral on its usefulness. While some interviewed patients found the app useful to improve self-awareness of their symptoms, many found the questionnaires too frequent, and one patient found that the lack of questionnaires about nonarticular symptoms was problematic. Some patients were not aware that their rheumatologist would use their reported data to determine visit timing (which was correct in some cases) or that their rheumatologist even had access to these data, which reduced their motivation to complete the questions. However, patients were largely enthusiastic about the idea of reducing the need for visits because of the time it could save them. Although some patients liked the idea of being called for earlier visits, many were skeptical that the data they reported in the app would be useful to their rheumatologists to help inform that decision.

### Implications and Comparison With Prior Work

Although we identified several rheumatologist- and patient-facing barriers, the participants who engaged in the intervention provided a proof of concept that this intervention has the potential to improve the efficiency of face-to-face visits by facilitating visit deferrals when patients have stable symptoms. Between-visit symptom monitoring has other benefits, such as helping patients be more aware of their symptoms so they can get help sooner and facilitating discussions of symptoms during visits [14,15,22-26]. This work provides early evidence that improving visit efficiency can be added to that list of potential benefits. Qualitative data and the wide range of dashboard usage by rheumatologists suggest that clinician engagement is likely the critical factor that determines the extent to which visit deferrals will be offered and patients find the app useful. Other clinically integrated digital interventions have also encountered this challenge [27-29].

Clinician-facing enhancements may include generating evidence of the benefits of further spacing out visits to improve buy-in, simplifying the patient rescheduling process, and making patients preferences for longer visit intervals available to them. Patient-facing enhancements may include personalizing the timing and topics of the questionnaires based on patient responses or specified preferences, allowing the patients to add notes to elaborate on their symptoms when the questionnaires do not adequately capture them, assessing patient interest in changing visits, and informing patients within the user interface of how their rheumatologists will use the data. These enhancements require additional design and user testing. This approach may also prove promising in other medical specialties such as pulmonology and cardiology, which will also require additional design effort.

To our knowledge, this is the first attempt to use data collected between visits to inform visit timing in the United States. Prior work in rheumatology has attempted to free up appointments using scheduled nurse phone calls but did not base decisions on longitudinally collected patient data [13]. A recent rheumatology trial in the Netherlands found that app-supported patient-initiated care achieved comparable clinical results with fewer rheumatology consults for patients with stable low disease activity [12].

### Limitations

This work has several limitations. First, the intervention was implemented in one academic health center with 11 rheumatologists who lacked direct incentives to be more efficient with visit timing. Other settings with different incentives may have had different experiences with the intervention. Second, the clinical logic for when an EHR inbox message was sent to the rheumatologists suggesting early or deferred visits was determined based on the expert opinion of one rheumatologist (DHS) with review and agreement by the participating study rheumatologists rather than through empirical evaluation. Therefore, the logic may have suggested changes to visit timing that were not appropriate or missed opportunities to change visits that were appropriate. Third, we were unable to comprehensively attribute an actual early or deferred visit to an app-generated message because we did not have data for when a provider initiated a patient outreach to offer an early or deferred visit in response to a study-generated message (that data is not recorded reliably or systematically in the EHR). Finally, responses to the rheumatologist survey may be influenced by social desirability bias [30]. Specifically, it is notable that none indicated that removing easier visits would be a barrier, and few selected barriers related to revenue and reimbursement, which may nonetheless be important factors to some clinicians.

### Conclusions

Informing visit timing systematically based on data collected from patients between specialty visits would, among other benefits, represent a fundamental advance in health care delivery—a move from one-size-fits-all visit timing to data-informed determinations that personalize care to individual patient needs. We examined how a PRO-focused mHealth app for RA might contribute to deferring unneeded follow-up visits. The app suggested many opportunities to defer visits, some of which resulted in rescheduling visits, though many did not. Based on our quantitative and qualitative results, clinicians had inconsistent buy-in, which patients perceived. The experience of the rheumatologists who did engage in the intervention and the patients who received the option to defer a visit provide a proof of concept that this intervention has the potential to succeed in reducing visit frequency if further enhanced based on user-centered design to improve clinician engagement and patient-perceived usefulness. It is also possible that in different health care settings, that is, nonacademic practices, or when used by clinicians practicing under different incentives (eg, capitation and performance measures relevant to access), such an app that allows for rescheduling patients may achieve greater uptake and produce significant improvements in access. Directly addressing clinician buy-in is likely the key factor and requires experimentation with different kinds of implementation strategies, such as stronger leadership support, audit feedback, and direct incentives. If successfully adopted, this type of intervention may reduce per-patient visit frequency, improve patient satisfaction, and improve access.

## Abbreviations

EHR: electronic health record
MAUQ: mHealth App Usability Questionnaire
MAUQ_E: mHealth App Usability Questionnaire for Ease of Use
MAUQ_U: mHealth App Usability Questionnaire for Usefulness
mHealth: mobile health
PRO: patient-reported outcome
RA: rheumatoid arthritis
SUS: System Usability Scale
